# Approaches to Managing Safety With Lenalidomide in Hematologic Malignancies

**DOI:** 10.6004/jadpro.2014.5.4.4

**Published:** 2014-07-01

**Authors:** Susan Blumel, Jackie Broadway-Duren

**Affiliations:** From University of Nebraska Medical Center, Omaha, Nebraska, and MD Anderson Cancer Center, Houston, Texas

## Abstract

Lenalidomide is an oral immunomodulatory agent approved in relapsed multiple myeloma with dexamethasone, for transfusion-dependent anemia in myelodysplastic syndrome associated with deletion 5q, and in relapsed/progressive mantle cell lymphoma following bortezomib. In recent clinical trials, lenalidomide has shown promising activity in hematologic malignancies, including chronic lymphocytic leukemia (CLL) and non-Hodgkin lymphoma (NHL). Starting doses and dosing schedules vary by malignancy, with lenalidomide started at a lower dose for CLL than for NHL or multiple myeloma. Certain adverse events (AEs) are common across tumor types (e.g., neutropenia, thrombocytopenia, fatigue), whereas others are more often associated with CLL patients (e.g., tumor lysis syndrome and tumor flare reaction). Effective management requires awareness of these differences as well as appropriate prophylaxis, monitoring, and treatment of AEs. This article reviews the efficacy and safety of lenalidomide in CLL and NHL, focusing on approaches for the advanced practitioner to improve patient quality of life through optimal management of side effects. With these steps, lenalidomide can be administered safely, at the best starting doses and with minimal dose interruptions or reductions across hematologic malignancies.

Lenalidomide (Revlimid) is an oral immunomodulatory agent approved in the United States in combination with dexamethasone for patients with multiple myeloma (MM) who have received one or more prior therapies and as a single agent for transfusion-dependent anemia due to low-/intermediate-1–risk myelodysplastic syndrome (MDS) associated with deletion 5q with/without additional cytogenetic abnormalities (Dimopoulos et al., 2007; Celgene, 2013; Weber et al., 2007). Its mechanisms of action involve multiple processes that depend on the tumor type and microenvironment to collectively reduce tumor cell proliferation and survival (Anderson, 2005; Chanan-Khan & Cheson, 2008; Hayashi et al., 2005; Kotla et al., 2009; Wu et al., 2008). The immunomodulatory properties of lenalidomide provide a basis for clinical investigations in patients with B-cell chronic lymphocytic leukemia (CLL) and non-Hodgkin lymphoma (NHL). This article reviews the efficacy and safety of lenalidomide in CLL and NHL, focusing on approaches for the advanced practitioner to improve patient quality of life through optimal management of side effects in patients receiving lenalidomide.

## CLINICAL STUDIES IN LYMPHOID MALIGNANCIES

**Relapsed/Refractory CLL** 

Early phase II investigations at Roswell Park Cancer Institute (RPCI) and M. D. Anderson Cancer Center (MDACC) focused on lenalidomide dose optimization in heavily pretreated patients with relapsed/refractory CLL for maximal activity without compromising safety (Chanan-Khan et al., 2006; Ferrajoli et al., 2008). Lenalidomide produced overall response rates (ORR) of 47% (21/45 patients, 9% complete response [CR], RPCI; Chanan-Khan et al., 2006) and 32% (14/44 patients, 7% CR, MDACC; Ferrajoli et al., 2008).

**Figure 1:**
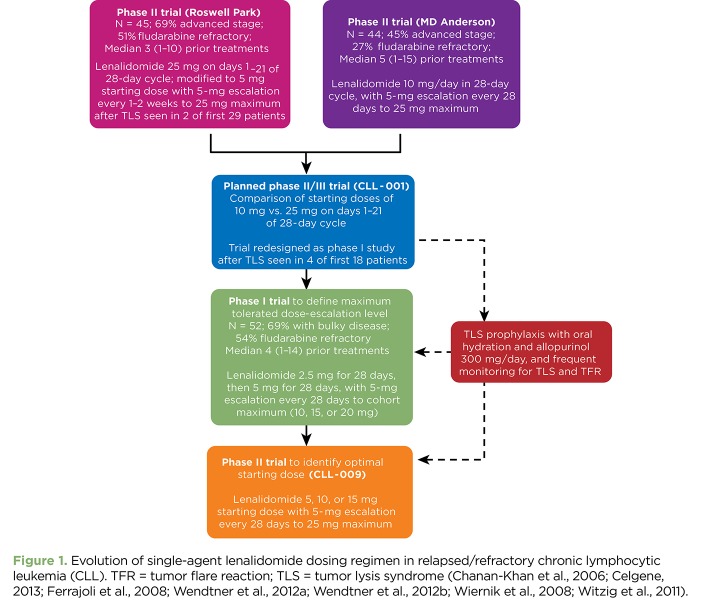
Evolution of single-agent lenalidomide dosing regimen in relapsed/refractory chronic lymphocytic leukemia (CLL). TFR = tumor flare reaction; TLS = tumor lysis syndrome (Chanan-Khan et al., 2006; Celgene, 2013; Ferrajoli et al., 2008; Wendtner et al., 2012a; Wendtner et al., 2012b; Wiernik et al., 2008; Witzig et al., 2011).

Tumor lysis syndrome (TLS) was observed in 2 of the first 29 patients who received 25 mg lenalidomide on days 1–21 of a 28-day cycle (Chanan-Khan et al., 2006), prompting lower initial doses with subsequent dose escalation (Figure 1; Chanan-Khan et al., 2006; Ferrajoli et al., 2008). Grade 3/4 adverse events (AEs) were mainly hematologic, and included neutropenia (70% patients [RPCI], 41% of treatment courses [MDACC]) and thrombocytopenia (45% of patients and 15% of treatment courses, respectively). Fatigue, diarrhea, rash, and tumor flare reactions (TFRs) were common nonhematologic AEs, although they were mostly grade 1/2 (Chanan-Khan et al., 2006; Ferrajoli et al., 2008).

Based on phase II studies, the CLL-001 phase II/III trial compared starting doses of 10 vs. 25 mg/day lenalidomide in patients with heavily pretreated, relapsed/refractory CLL (see Figure 1; Wendtner et al., 2012b). Patients had a median age of 65 years; 69% had bulky lymphadenopathy (> 5 cm), and 48% had high-risk genomic abnormalities. Four serious cases of TLS were observed in the first 18 patients, leading to a protocol amendment to identify the maximum tolerated dose (MTD, defined as the highest dose of a treatment that does not cause unacceptable side effects) escalation level with lower initial lenalidomide, added TLS prophylaxis, increased TLS/TFR monitoring, and the exclusion of any patients with severe renal dysfunction, who were defined as those having a history of renal failure that required dialysis (Moutouh-de Parseval, Weiss, DeLap, Knight, & Zeldis, 2007).

Dose escalation from 2.5 mg/day lenalidomide, increasing in 5-mg increments every 28 days, achieved safe titration to 20 mg/day without reaching the MTD (Wendtner et al., 2012b). The most common grade 3/4 AEs were neutropenia (65%), thrombocytopenia (33%), and pneumonia (21%). The occurrence of 4% TLS and 44% TFR (10% grade 3) was successfully managed with treatment (e.g., nonsteroidal anti-inflammatory agents or corticosteroids) and/or temporary treatment interruption. A total of 58% of patients experienced ≥ 1 dose reduction/interruption due to AEs. Six patients reached the maximum dose of 20 mg; other dose levels of 15 mg (n = 10), 10 mg (n = 14), 5 mg (n = 6), and 2.5 mg (n = 16) were also achieved. Six patients (12%) achieved partial responses with 10 to 20 mg lenalidomide. Thirty patients (58%) had stable disease, including 7 at a maximum 2.5 mg. Median progression-free survival (PFS) was 24.1 weeks for all patients and 42.1 weeks for responders.

This conservative dose-escalation approach with lenalidomide for heavily pretreated, bulky, high-risk CLL patients demonstrated safe titration from an initial dose of 2.5 mg up to 20 mg. Moreover, the use of TLS prophylaxis and monitoring may facilitate more rapid dose escalation or higher starting doses in future studies.

Based on these findings, a randomized, double-blind phase II trial (CLL-009; ClinicalTrials.gov Identifier NCT00963105) was initiated in relapsed/refractory CLL, with lenalidomide at 5, 10, or 15 mg daily (28-day cycles), with dose escalation in 5-mg increments every 28 days as tolerated to 25 mg/day (Wendtner et al., 2012a). The most common grade 3/4 AEs seen in 104 patients were neutropenia (67%), thrombocytopenia (38%), pneumonia (14%), TFR (14%), and fatigue (12%). Grade 3 TLS occurred in four patients (1 at 5 mg/day, 3 at 15 mg/day). Dose escalation to 25 mg/day was achieved in 24% of patients; the mean dose administered was 12 mg/day. The overall response rate was 38% (102/104 evaluable patients), including three patients with CR (3%).

## B-Cell NHL

**Indolent NHL** 

Indolent lymphomas comprise a group of incurable and generally slow-growing entities, of which follicular lymphoma (FL), marginal zone lymphoma, and small lymphocytic lymphoma (SLL) are the most common (Armitage &s Weisenburger, 1998; Lunning & Vose, 2012). The phase II NHL-001 study of lenalidomide 25 mg/day on days 1 through 21 every 28 days produced a modest 23% ORR (10/43 patients), including 7% CR/CR unconfirmed (CRu) in heavily pretreated patients with refractory indolent lymphoma (Witzig et al., 2009). Median PFS was 4.4 months, and median duration of response (DOR) was > 16.5 months, with 7/10 responses ongoing at 15 to 28 months. Adverse events were predictable; the most common grade 3/4 AEs were neutropenia (46%) and thrombocytopenia (19%). Tumor flare reactions occurred in 3/18 SLL patients and 1 FL patient but were not correlated with response. Studies continue to be conducted in indolent lymphomas (ClinicalTrials.gov Identifiers NCT00695786, NCT01938001, NCT01316523, NCT01996865, and NCT01476787).

**Mantle Cell Lymphoma** 

Mantle cell lymphoma (MCL) is an aggressive subtype of NHL initially treated with induction chemoimmunotherapy, but with relatively short duration and poor prognosis upon relapse (Habermann et al., 2009). The NHL-002 and NHL-003 trials included 15 and 57 MCL patients receiving lenalidomide who achieved ORRs of 53% (20% CR) and 42% (21% CR), respectively (Habermann et al., 2009; Witzig et al., 2011). Across the studies, grade 3/4 neutropenia and thrombocytopenia were reported in approximately 40% and 20% of patients, respectively. Results from the recent prospective phase II MCL-001 study (EMERGE) confirmed these findings in 134 heavily pretreated MCL patients who were relapsed/refractory to bortezomib (Goy et al., 2013). Mantle cell lymphoma patients showed a 28% ORR (7.5% CR/CRu), with a durable median DOR of 16.6 months.

The most common grade 3/4 AEs were neutropenia (43%), thrombocytopenia (28%), and anemia (11%). Rash was reported in 30 patients (22%; grade 1/2 in 28 patients) and was manageable with antihistamines or low-dose steroids. Grade 1/2 TFR was reported in 13 patients (10%); there were no reports of TLS. The EMERGE study demonstrated predictable safety and durable activity of lenalidomide in heavily pretreated patients with advanced- stage relapsed/refractory MCL post-bortezomib, regardless of tumor burden, prior autologous stem cell transplantation (ASCT), or number of prior therapies (Goy et al., 2013).

**Diffuse Large B-Cell Lymphoma** 

Diffuse large B-cell lymphoma (DLBCL), the most common form of NHL, has an aggressive clinical course with poor prognosis after first relapse. In a pooled analysis of patients from the NHL-002 and NHL-003 studies, 35/134 relapsed/refractory DLBCL patients achieved a 26% ORR (12% CR) and median 6.0-month DOR (10.4 months for responders). Consistent with other studies of lenalidomide, neutropenia (36%) and thrombocytopenia (21%) were the most common grade 3/4 AEs.

Diffuse large B-cell lymphoma can be divided into subgroups with distinct characteristics and prognoses based on gene expression profiling (Hernandez-Ilizaliturri et al., 2011). In a retrospective analysis of 40 relapsed/refractory DLBCL patients, non–germinal center B-cell (non-GCB)–like vs. germinal center B-cell (GCB)–like patients treated with lenalidomide showed similar overall survival but significantly higher ORR (53% vs. 9%, *p* = .006), CR (24% vs. 4%), and median PFS (6.2 vs. 1.7 months, *p* = .004), respectively (Hernandez-Ilizaliturri et al., 2011). These findings remain to be validated in future studies.

## SIDE-EFFECT MONITORING AND MANAGEMENT IN LYMPHOID MALIGNANCIES

Hematologic toxicities consistently comprise the most common grade 3/4 AEs with lenalidomide, and nonhematologic toxicity varies across malignancies (Table 1). Suggested monitoring and treatment recommendations for the most common AEs are based on the regulatory-approved indications in MM/MDS and clinical experiences with CLL and NHL (Table 2; Celgene, 2013). A closer look at nonhematologic AEs is outlined below.

**Table 1:**
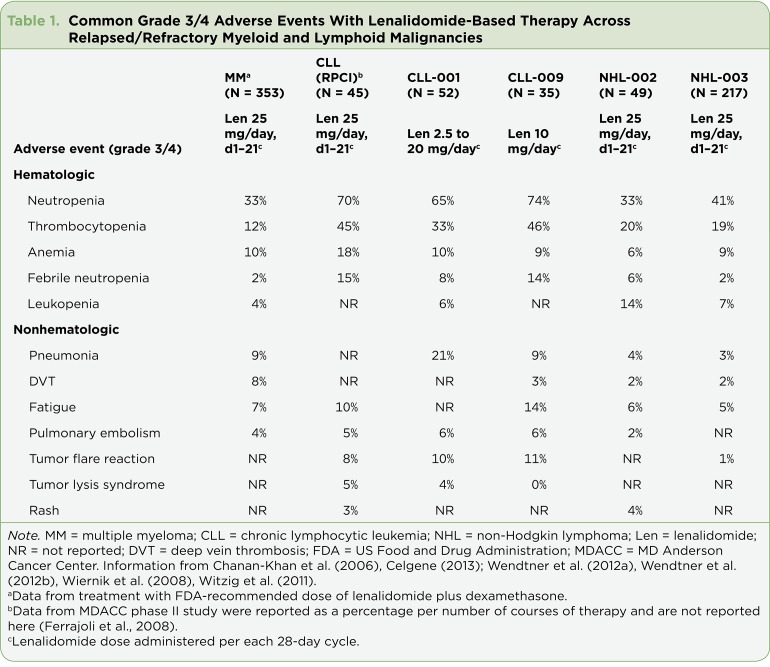
Common Grade 3/4 Adverse Events With Lenalidomide-Based Therapy Across Relapsed/Refractory Myeloid and Lymphoid Malignancies

**Table 2:**
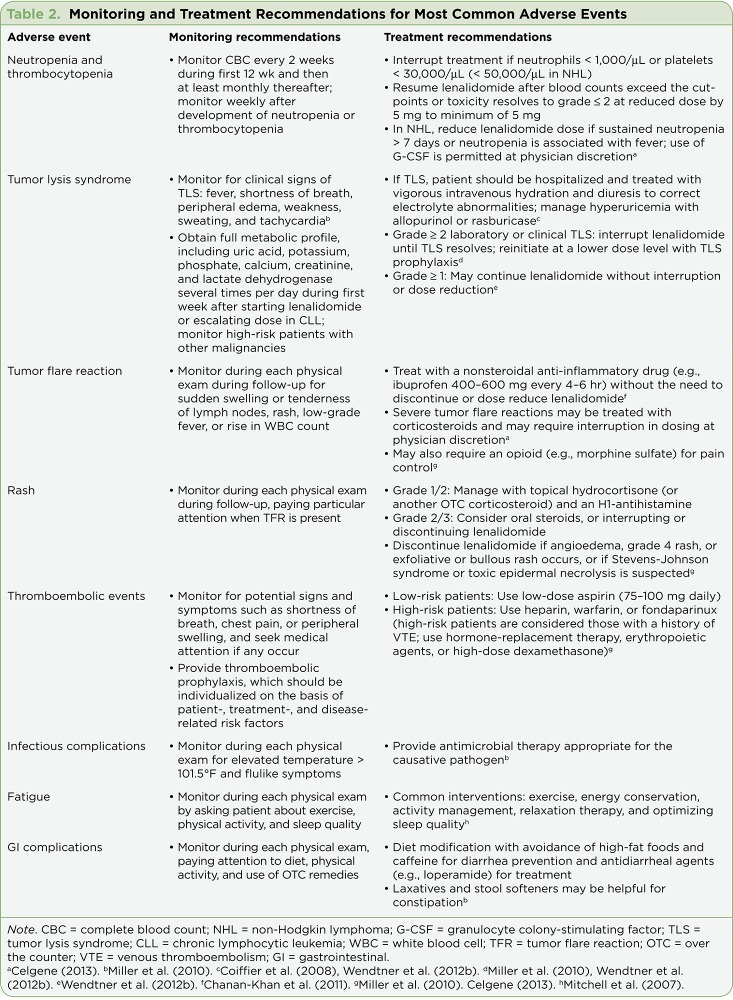
Monitoring and Treatment Recommendations for Most Common Adverse Events

**Tumor Lysis Syndrome** 

Tumor lysis syndrome is a group of metabolic derangements that may occur when malignant cells are rapidly killed, causing a massive release of intracellular metabolites into the bloodstream (Coiffier, Altman, Pui, Younes, & Cairo, 2008). Symptoms include hyperuricemia, hyperkalemia, hyperphosphatemia, hypocalcemia, cardiac arrhythmia, and uremia, which may lead to renal dysfunction and potentially acute renal failure. Risk factors include hematologic malignancies, bulky disease (> 10 cm), preexisting renal insufficiency, elevated baseline serum/plasma uric acid level > 7.5 mg/dL, dehydration, elevated lactic dehydrogenase level (> 2 times upper limit of normal), and rapid cytoreduction following treatment (Coiffier et al., 2008; McGraw, 2008).

Tumor lysis syndrome was seen in 7/260 CLL patients (3%) receiving lenalidomide in a review of the Celgene Corporation database conducted in 2007, with all cases developing during the first 15 days of treatment (Moutouh-de Parseval et al., 2007). Acute renal failure and/or cardiac arrhythmia were seen in 3/7 patients. Slow dose titration and TLS prophylaxis were subsequently initiated in the CLL-001 trial, including oral hydration (to promote urinary excretion of uric acid and phosphate) and allopurinol 300 mg daily (to prevent xanthine and hypoxanthine conversion into uric acid) 3 days prior to lenalidomide and continued for 3 cycles (Wendtner et al., 2012b). Tumor lysis syndrome prophylaxis with allopurinol 300 mg daily was provided on days 1 through 14 of the first cycle in a subsequent study of lenalidomide in CLL patients (Badoux et al., 2011). These practices were carried over into the CLL-009 study to enable identification of the optimal starting dose of lenalidomide in CLL patients (Wendtner et al., 2012b).

**Tumor Flare Reaction** 

Tumor flare reaction in CLL is characterized by a sudden and/or tender enlargement of the lymph nodes and/or spleen, often in association with low-grade fever and rash, and sometimes bone pain or increased white blood cells (Chanan-Khan et al., 2006; Ferrajoli et al., 2008). It is important for advanced practitioners to recognize TFR as a possible complication of therapy, as the associated signs and symptoms may be mistaken for disease progression (Table 2).

Tumor flare reaction developed in 44% (10% grade 3) of CLL patients in the CLL-001 trial (Wendtner et al., 2012b). In the MDACC phase II trial, any-grade TFR was higher among patients with lymph nodes > 5 cm (53% vs. 15% for patients with 5 cm nodes; Ferrajoli et al., 2008). In the RPCI phase II study, TFR incidence (58% overall, 8% grade 3/4) was associated with advanced-stage CLL and younger age, but not with bulky disease (Chanan-Khan et al., 2006, 2011). Severe, life-threatening TFR that necessitated hospitalization was reported in four patients with relapsed/refractory CLL who received lenalidomide at a starting dose of 25 mg (Andritsos et al., 2008). Tumor flare reaction was reported in 14% of patients overall in the CLL-009 study, at a similar incidence regardless of the starting dose of lenalidomide (5, 10, or 15 mg/day; Wendtner et al., 2012a).

Tumor flare reaction usually occurs during the first treatment cycle, with a median time to onset of 6 days (range, 0–56) and a median time to resolution of 14 days (95% CI, 10-26), and the intensity of TFR may be positively correlated with achieving a CR with lenalidomide in CLL patients (Chanan-Khan et al., 2011). Prophylaxis with low- dose oral prednisone (20 mg for 5 days followed by 10 mg for 5 days) decreased severity and delayed onset but did not reduce the incidence and may slow resolution (Chanan-Khan et al., 2011). Tumor flare reaction has also been reported mainly within the first cycle of lenalidomide in relapsed MCL patients, including 13/134 patients (10%, all grade 1/2) in the MCL-001 study and 4/26 patients (15%; 3 grade 2) reported by Eve and Rule (Eve & Rule, 2010; Goy et al., 2013).

**Rash** 

Grade 1/2 rash is relatively common with lenalidomide in CLL and NHL, often presenting as generalized pruritic, macular, and/or raised erythema (Miller, Musial, Whitworth, & Chanan-Khan, 2010). In clinical studies, rash was reported in 40% of CLL patients and approximately 30% of NHL patients; grade 3 events were uncommon at < 5% (Chanan-Khan et al., 2006; Ferrajoli et al., 2008; Wendtner et al., 2012b; Wiernik et al., 2008; Witzig et al., 2011). Rash was also observed in 46% of patients with indolent lymphoma who received combination lenalidomide and rituximab therapy in a phase II study; most cases were grade 1/2 (Nelson et al., 2012). Rash may be associated with TFR or caused by treatment.

Treatment of rash is directed by severity of symptoms and may include observation, oral or topical antihistamines, oral or topical steroids, or in severe cases, discontinuation of lenalidomide. Hypersensitivity reactions include rare cases of Stevens-Johnson syndrome and toxic epidermal necrolysis (0.02% based on postmarketing reports of lenalidomide use for MM, myelofibrosis, and amyloidosis), in which cytotoxicity causes separation of the epidermis from the dermis (Castaneda, Brandenburg, Bwire, Burton, & Zeldis, 2009).

**Deep Vein Thrombosis and Pulmonary Embolism** 

Lenalidomide may carry a thromboembolic risk in CLL and NHL. Grade 3/4 pulmonary embolism was observed in 3/52 patients (6%) in the CLL-001 trial (Wendtner et al., 2012b), and grade 3 deep vein thrombosis occurred in 5/217 patients (2%) in the NHL-003 trial (Witzig et al., 2011). Although rare, thrombotic events are potentially life threatening. Consideration of daily prophylactic low-dose aspirin is warranted in patients not currently receiving anticoagulant therapy (e.g., warfarin; Miller et al., 2010).

**Other Adverse Events** 

Grade 3/4 infections (e.g., pneumonia) were reported in CLL, likely reflective of prior treatment and the general immunocompromised nature of the disease (Miller et al., 2010). Grade 3/4 infection-related AEs occurred in 40% of patients in the CLL-001 study, including 21% with pneumonia (Wendtner et al., 2012b). Severe infections complicated 6% of treatment cycles in the phase II MDACC trial (Ferrajoli et al., 2008) but were less common in the RPCI phase II trial at 4% (Chanan-Khan et al., 2006). Non-Hodgkin lymphoma patients were less prone to infections during treatment: 4% grade 3/4 pneumonitis in NHL-002 and 3% grade 3/4 pneumonia in NHL-003; see Table 1 (Wiernik et al., 2008; Witzig et al., 2011).

The incidence of predominantly grade 1/2 fatigue is common in both CLL and NHL. Fatigue increased from baseline following initiation of lenalidomide in CLL and was present at baseline (29%) and during treatment (73%) in the RPCI study (Chanan-Khan et al., 2006). Four patients had grade 3/4 fatigue, which resolved completely in two cases during continued lenalidomide. Any-grade fatigue was common in NHL patients in both the NHL-002 (49% overall, 6% grade 3) and NHL-003 (28% overall, 5% grade 3) studies without the need for dose interruption or reduction; monitoring/management recommendations are outlined in Table 2 (Chanan-Khan et al., 2006; Wiernik et al., 2008; Witzig et al., 2011).

Diarrhea and constipation are the most common gastrointestinal complications associated with lenalidomide (Chanan-Khan et al., 2006; Ferrajoli et al., 2008; Wiernik et al., 2008; Witzig et al., 2011). Common interventions (e.g., diet modification and laxative use) have effectively managed these AEs (Miller et al., 2010).

**Second Primary Malignancies After Lenalidomide Use** 

Patients with cancer are at increased risk of developing second primary malignancies (SPMs), which are influenced by multiple factors, including age and prolonged exposure to chemotherapy (especially alkylating agents) and radiation (Barista et al., 2002; Decaudin et al., 2000; Dimopoulos et al., 2012; Palumbo, Freeman, Weiss, & Fenaux, 2012; Romaguera et al., 2005). Limited SPM data are reported, with the majority of information in relapsed/refractory MM.

A retrospective review of 11 lenalidomide studies in relapsed/refractory MM showed 52 invasive SPMs in 3,846 patients, for an overall incidence rate of 2.08 per 100 patient-years (Altekruse et al., 2010; Dimopoulos et al., 2012; Palumbo et al., 2012). In more recent studies in patients with relapsed/refractory MCL receiving lenalidomide, 3/134 (2%) patients in MCL-001 and 2/57 (3.5%) in NHL-003 reported invasive SPMs (Goy et al., 2013; Zinzani et al., 2013). To date, the incidence of SPMs with lenalidomide treatment is comparable to the rate of 2.1 per 100 patient-years expected in the general population of older adults (Altekruse et al., 2010).

**Risk Counseling** 

Counseling and education of patients regarding potentially life-threatening risks should be conducted at regular intervals before and throughout treatment. Patients must be informed of significant neutropenia and thrombocytopenia risks that may require dose modification, transfusions, and/or growth factor administration. Patients must also be informed regarding thromboembolic risks and instructed to immediately report symptoms such as shortness of breath, difficulty breathing, chest pain, or swelling of the extremities.

Prevention of fetal risk is an educational priority for patients receiving lenalidomide, given that it is a thalidomide analog. Females of childbearing potential should have two negative pregnancy tests before starting treatment and must use two forms of birth control until 4 weeks after treatment discontinuation. Males taking lenalidomide must use contraceptives during any sexual contact with a female with childbearing potential, and they must refrain from donating sperm.

Lenalidomide, marketed as Revlimid, is only available through the Revlimid Risk Evaluation and Mitigation Strategy (REMS), a restricted distribution program. Only certified prescribers and pharmacies can prescribe and dispense lenalidomide to patients who are enrolled and meet all the conditions of the REMS program.

## DOSING SCHEDULES AND OPTIMIZATION IN MM, CLL, AND NHL

**Figure 2:**
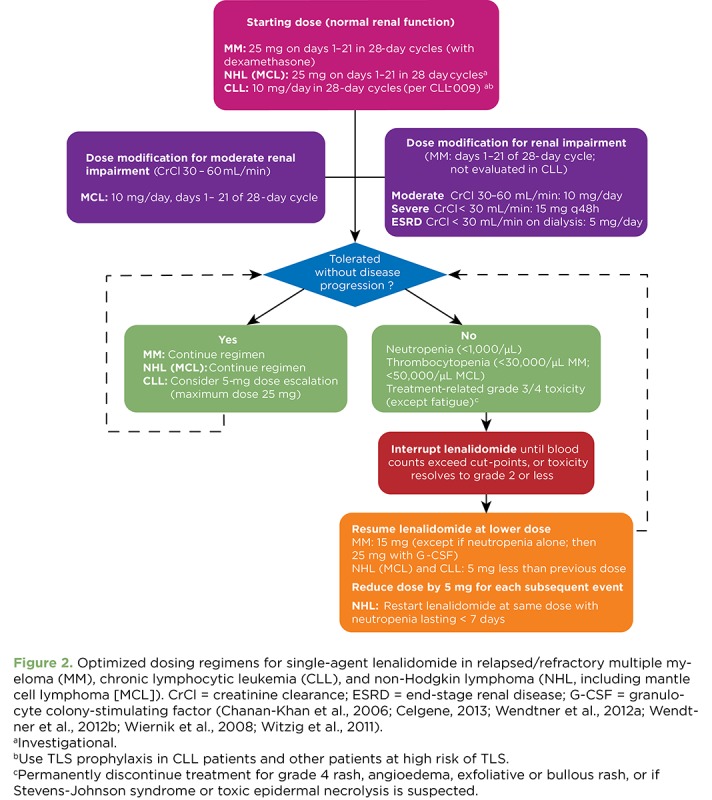
Optimized dosing regimens for single-agent lenalidomide in relapsed/refractory multiple myeloma (MM), chronic lymphocytic leukemia (CLL), and non-Hodgkin lymphoma (NHL, including mantle cell lymphoma [MCL]). CrCl = creatinine clearance; ESRD = end-stage renal disease; G-CSF = granulocyte colony-stimulating factor (Chanan-Khan et al., 2006; Celgene, 2013; Wendtner et al., 2012a; Wendtner et al., 2012b; Wiernik et al., 2008; Witzig et al., 2011).

Current dosing schedules are outlined in Figure 2. Dose adjustments are recommended for renal impairment initially or from resultant cytopenia/other grade 3/4 AEs. The dosing schedule in MM provided a basis for that in relapsed/refractory NHL, as shown in the NHL-001, NHL-002, and NHL-003 studies (Wiernik et al., 2008; Witzig et al., 2011; Witzig et al., 2009). A lower initial lenalidomide dose of 20 mg/day may be needed to minimize toxicity when used in combination, as shown in relapsed/refractory MCL with lenalidomide plus rituximab (Wang et al., 2012). Patients with CLL require a lower starting dose (e.g., 5–10 mg/day) to minimize TLS/TFR risks, with dose escalation every 28 days as tolerated (Wendtner et al., 2012a).

## DISCUSSION

Recent clinical trials support the activity of lenalidomide in lymphoid malignancies, including CLL and NHL, and show that dose levels and certain toxicities differ across cancer types. The lenalidomide schedule used in MM also appears to be appropriate for NHL, but a lower starting dose was used in CLL to minimize certain AEs. Hematologic toxicity, mainly neutropenia and thrombocytopenia, is common across malignancies; therefore regular monitoring is recommended. Tumor flare reaction and TLS are potentially serious toxicities seen in CLL. Other AEs, such as rash, fatigue, diarrhea, and infection, can generally be managed with conventional strategies.

Advanced practitioners are pivotal in providing the appropriate prophylaxis, patient counseling, monitoring, and treatment for common toxicities that enables lenalidomide to be administered in a safe manner at optimal dose levels as an active therapy in hematologic malignancies. 

## Acknowledgments

Medical writing assistance in the preparation of this manuscript was provided by Barry M. Weichman, PhD, and Julie Kern, PhD, CMPP, with Bio Connections LLC and funded by Celgene Corporation. The authors are fully responsible for content and editorial decisions for this manuscript.

## References

[1] Altekruse S. F., Kosary C. L., Krapcho M., Neyman N., Aminou R., Waldron W., Edwards B. K. (2010). *SEER cancer statistics review, 1975-2007*.

[2] Anderson Kenneth C (2005). Lenalidomide and thalidomide: mechanisms of action--similarities and differences.. *Seminars in hematology*.

[3] Andritsos Leslie A, Johnson Amy J, Lozanski Gerard, Blum William, Kefauver Cheryl, Awan Farrukh, Smith Lisa L, Lapalombella Rosa, May Sarah E, Raymond Chelsey A, Wang Da-Sheng, Knight Robert D, Ruppert Amy S, Lehman Amy, Jarjoura David, Chen Ching-Shih, Byrd John C (2008). Higher doses of lenalidomide are associated with unacceptable toxicity including life-threatening tumor flare in patients with chronic lymphocytic leukemia.. *Journal of clinical oncology : official journal of the American Society of Clinical Oncology*.

[4] Armitage J O, Weisenburger D D (1998). New approach to classifying non-Hodgkin’s lymphomas: clinical features of the major histologic subtypes. Non-Hodgkin’s Lymphoma Classification Project.. *Journal of clinical oncology : official journal of the American Society of Clinical Oncology*.

[5] Badoux Xavier C, Keating Michael J, Wen Sijin, Lee Bang-Ning, Sivina Mariela, Reuben James, Wierda William G, O’Brien Susan M, Faderl Stefan, Kornblau Steven M, Burger Jan A, Ferrajoli Alessandra (2011). Lenalidomide as initial therapy of elderly patients with chronic lymphocytic leukemia.. *Blood*.

[6] Barista I, Cabanillas F, Romaguera J E, Khouri I F, Yang Y, Smith T L, Strom S S, Medeiros L J, Hagemeister F B (2002). Is there an increased rate of additional malignancies in patients with mantle cell lymphoma?. *Annals of oncology : official journal of the European Society for Medical Oncology / ESMO*.

[7] Castaneda Carmen P, Brandenburg Nancy A, Bwire Robert, Burton Graham H, Zeldis Jerome B (2009). Erythema multiforme/Stevens-Johnson syndrome/toxic epidermal necrolysis in lenalidomide-treated patients.. *Journal of clinical oncology : official journal of the American Society of Clinical Oncology*.

[8] Celgene (2013). *Revlimid (lenalidomide) package insert*.

[9] Chanan-Khan Asher A, Cheson Bruce D (2008). Lenalidomide for the treatment of B-cell malignancies.. *Journal of clinical oncology : official journal of the American Society of Clinical Oncology*.

[10] Chanan-Khan Asher, Miller Kena C, Lawrence David, Padmanabhan Swaminathan, Miller Austin, Hernandez-Illatazurri Francisco, Czuczman Myron S, Wallace Paul K, Zeldis Jerome B, Lee Kelvin (2011). Tumor flare reaction associated with lenalidomide treatment in patients with chronic lymphocytic leukemia predicts clinical response.. *Cancer*.

[11] Chanan-Khan Asher, Miller Kena C, Musial Laurie, Lawrence David, Padmanabhan Swaminathan, Takeshita Kenichi, Porter Carl W, Goodrich David W, Bernstein Zale P, Wallace Paul, Spaner David, Mohr Alice, Byrne Catriona, Hernandez-Ilizaliturri Francisco, Chrystal Cynthia, Starostik Petr, Czuczman Myron S (2006). Clinical efficacy of lenalidomide in patients with relapsed or refractory chronic lymphocytic leukemia: results of a phase II study.. *Journal of clinical oncology : official journal of the American Society of Clinical Oncology*.

[12] Coiffier Bertrand, Altman Arnold, Pui Ching-Hon, Younes Anas, Cairo Mitchell S (2008). Guidelines for the management of pediatric and adult tumor lysis syndrome: an evidence-based review.. *Journal of clinical oncology : official journal of the American Society of Clinical Oncology*.

[13] Czuczman M., Vose J., Zinzani P. L., Reeder C., Buckstein R., Haioun C., Witzig T. (2010). Lenalidomide monotherapy is clinically active in patients with relapsed/refractory diffuse large B-cell lymphoma (DLBCL): A pooled analysis of data form phase II studies (NHL-002/003) [Abstract 574].. * Haematologica*.

[14] Decaudin D, Brousse N, Brice P, Haioun C, Bourhis J H, Morel P, Van Hoof A, Souleau B, Quesnel B, Gisselbrecht C (2000). Efficacy of autologous stem cell transplantation in mantle cell lymphoma: a 3-year follow-up study.. *Bone marrow transplantation*.

[15] Dimopoulos Meletios A, Richardson Paul G, Brandenburg Nancy, Yu Zhinuan, Weber Donna M, Niesvizky Ruben, Morgan Gareth J (2012). A review of second primary malignancy in patients with relapsed or refractory multiple myeloma treated with lenalidomide.. *Blood*.

[16] Dimopoulos Meletios, Spencer Andrew, Attal Michael, Prince H Miles, Harousseau Jean-Luc, Dmoszynska Anna, San Miguel Jesus, Hellmann Andrzej, Facon Thierry, Foà Robin, Corso Alessandro, Masliak Zvenyslava, Olesnyckyj Marta, Yu Zhinuan, Patin John, Zeldis Jerome B, Knight Robert D (2007). Lenalidomide plus dexamethasone for relapsed or refractory multiple myeloma.. *The New England journal of medicine*.

[17] Eve Heather E, Rule Simon A J (2010). Lenalidomide-induced tumour flare reaction in mantle cell lymphoma.. *British journal of haematology*.

[18] Ferrajoli Alessandra, Lee Bang-Ning, Schlette Ellen J, O’Brien Susan M, Gao Hui, Wen Sijin, Wierda William G, Estrov Zeev, Faderl Stefan, Cohen Evan N, Li Changping, Reuben James M, Keating Michael J (2008). Lenalidomide induces complete and partial remissions in patients with relapsed and refractory chronic lymphocytic leukemia.. *Blood*.

[19] Goy Andre, Sinha Rajni, Williams Michael E, Kalayoglu Besisik Sevgi, Drach Johannes, Ramchandren Radhakrishnan, Zhang Lei, Cicero Sherri, Fu Tommy, Witzig Thomas E (2013). Single-agent lenalidomide in patients with mantle-cell lymphoma who relapsed or progressed after or were refractory to bortezomib: phase II MCL-001 (EMERGE) study.. *Journal of clinical oncology : official journal of the American Society of Clinical Oncology*.

[20] Habermann Thomas M, Lossos Izidore S, Justice Glen, Vose Julie M, Wiernik Peter H, McBride Kyle, Wride Kenton, Ervin-Haynes Annette, Takeshita Kenichi, Pietronigro Dennis, Zeldis Jerome B, Tuscano Joseph M (2009). Lenalidomide oral monotherapy produces a high response rate in patients with relapsed or refractory mantle cell lymphoma.. *British journal of haematology*.

[21] Hayashi Toshiaki, Hideshima Teru, Akiyama Masaharu, Podar Klaus, Yasui Hiroshi, Raje Noopur, Kumar Shaji, Chauhan Dharminder, Treon Steven P, Richardson Paul, Anderson Kenneth C (2005). Molecular mechanisms whereby immunomodulatory drugs activate natural killer cells: clinical application.. *British journal of haematology*.

[22] Hernandez-Ilizaliturri Francisco J, Deeb George, Zinzani Pier L, Pileri Stefano A, Malik Farhana, Macon William R, Goy Andre, Witzig Thomas E, Czuczman Myron S (2011). Higher response to lenalidomide in relapsed/refractory diffuse large B-cell lymphoma in nongerminal center B-cell-like than in germinal center B-cell-like phenotype.. *Cancer*.

[23] Kotla V., Goel S., Nischal S., Heuck C., Vivek K., Das B., Verma A. (2009). Mechanism of action of lenalidomide in hematological malignancies. *Journal of Hematology & Oncology*.

[24] Lunning Matthew A, Vose Julie M (2012). Management of indolent lymphoma: where are we now and where are we going.. *Blood reviews*.

[25] McGraw Beth (2008). At an increased risk: tumor lysis syndrome.. *Clinical journal of oncology nursing*.

[26] Miller Kena C, Musial Laurie, Whitworth Amy, Chanan-Khan Asher (2010). Management of patients with chronic lymphocytic leukemia treated with lenalidomide.. *Clinical journal of oncology nursing*.

[27] Mitchell Sandra A, Beck Susan L, Hood Linda Edwards, Moore Karen, Tanner Ellen R (2007). Putting evidence into practice: evidence-based interventions for fatigue during and following cancer and its treatment.. *Clinical journal of oncology nursing*.

[28] Moutouh-de Parseval L. A., Weiss L., DeLap R. J., Knight R. D., Zeldis J. B. (2007). Tumor lysis syndrome/tumor flare reaction in lenalidomide-treated chronic lymphocytic leukemia.. *Journal of Clinical Oncology*.

[29] Nelson K., Samaniego F., Hagemeister F., Lacerte L., Kwak L. W., Neelapu S., Fowler N. (2012). Dermatologic side effects of lenalidomide and rituximab in indolent lymphoma.2012. *Pan Pacific Lymphoma Conference*.

[30] Palumbo Antonio, Freeman John, Weiss Lilia, Fenaux Pierre (2012). The clinical safety of lenalidomide in multiple myeloma and myelodysplastic syndromes.. *Expert opinion on drug safety*.

[31] Romaguera Jorge E, Fayad Luis, Rodriguez Maria A, Broglio Kristine R, Hagemeister Frederick B, Pro Barbara, McLaughlin Peter, Younes Anas, Samaniego Felipe, Goy Andre, Sarris Andreas H, Dang Nam H, Wang Michael, Beasley Virginia, Medeiros L Jeffrey, Katz Ruth L, Gagneja Harish, Samuels Barry I, Smith Terry L, Cabanillas Fernando F (2005). High rate of durable remissions after treatment of newly diagnosed aggressive mantle-cell lymphoma with rituximab plus hyper-CVAD alternating with rituximab plus high-dose methotrexate and cytarabine.. *Journal of clinical oncology : official journal of the American Society of Clinical Oncology*.

[32] Wang Michael, Fayad Luis, Wagner-Bartak Nicolaus, Zhang Liang, Hagemeister Fredrick, Neelapu Sattva S, Samaniego Felipe, McLaughlin Peter, Fanale Michelle, Younes Anas, Cabanillas Fernando, Fowler Nathan, Newberry Kate J, Sun Luhong, Young Ken H, Champlin Richard, Kwak Larry, Feng Lei, Badillo Maria, Bejarano Maria, Hartig Kimberly, Chen Wendy, Chen Yiming, Byrne Catriona, Bell Neda, Zeldis Jerome, Romaguera Jorge (2012). Lenalidomide in combination with rituximab for patients with relapsed or refractory mantle-cell lymphoma: a phase 1/2 clinical trial.. *The Lancet. Oncology*.

[33] Weber Donna M, Chen Christine, Niesvizky Ruben, Wang Michael, Belch Andrew, Stadtmauer Edward A, Siegel David, Borrello Ivan, Rajkumar S Vincent, Chanan-Khan Asher Alban, Lonial Sagar, Yu Zhinuan, Patin John, Olesnyckyj Marta, Zeldis Jerome B, Knight Robert D (2007). Lenalidomide plus dexamethasone for relapsed multiple myeloma in North America.. *The New England journal of medicine*.

[34] Wendtner C., Hallek M., Fraser G., Michallet A. S., Hillmen  P., Duerig J., Chanan-Khan A. (2012a). Updated interim results of the safety and efficacy of different lenalidomide starting dose regimens in patients with relapsed or refractory (rel/ref) chronic lymphocytic leukemia (CLL) (CC-5013-CLL-009 Study) [Abstract 3925].. *Blood (ASH Annual Meeting Abstracts)*.

[35] Wendtner Clemens-Martin, Hillmen Peter, Mahadevan Daruka, Bühler Andreas, Uharek Lutz, Coutré Steven, Frankfurt Olga, Bloor Adrian, Bosch Francesc, Furman Richard R, Kimby Eva, Gribben John G, Gobbi Marco, Dreisbach Luke, Hurd David D, Sekeres Mikkael A, Ferrajoli Alessandra, Shah Sheetal, Zhang Jennie, Moutouh-de Parseval Laure, Hallek Michael, Heerema Nyla A, Stilgenbauer Stephan, Chanan-Khan Asher A (2012b). Final results of a multicenter phase 1 study of lenalidomide in patients with relapsed or refractory chronic lymphocytic leukemia.. *Leukemia & lymphoma*.

[36] Wiernik Peter H, Lossos Izidore S, Tuscano Joseph M, Justice Glen, Vose Julie M, Cole Craig E, Lam Wendy, McBride Kyle, Wride Kenton, Pietronigro Dennis, Takeshita Kenichi, Ervin-Haynes Annette, Zeldis Jerome B, Habermann Thomas M (2008). Lenalidomide monotherapy in relapsed or refractory aggressive non-Hodgkin’s lymphoma.. *Journal of clinical oncology : official journal of the American Society of Clinical Oncology*.

[37] Witzig T E, Vose J M, Zinzani P L, Reeder C B, Buckstein R, Polikoff J A, Bouabdallah R, Haioun C, Tilly H, Guo P, Pietronigro D, Ervin-Haynes A L, Czuczman M S (2011). An international phase II trial of single-agent lenalidomide for relapsed or refractory aggressive B-cell non-Hodgkin’s lymphoma.. *Annals of oncology : official journal of the European Society for Medical Oncology / ESMO*.

[38] Witzig Thomas E, Wiernik Peter H, Moore Timothy, Reeder Craig, Cole Craig, Justice Glen, Kaplan Henry, Voralia Michael, Pietronigro Dennis, Takeshita Kenichi, Ervin-Haynes Annette, Zeldis Jerome B, Vose Julie M (2009). Lenalidomide oral monotherapy produces durable responses in relapsed or refractory indolent non-Hodgkin’s Lymphoma.. *Journal of clinical oncology : official journal of the American Society of Clinical Oncology*.

[39] Wu Lei, Adams Mary, Carter Troy, Chen Roger, Muller George, Stirling David, Schafer Peter, Bartlett J Blake (2008). lenalidomide enhances natural killer cell and monocyte-mediated antibody-dependent cellular cytotoxicity of rituximab-treated CD20+ tumor cells.. *Clinical cancer research : an official journal of the American Association for Cancer Research*.

[40] Zinzani P L, Vose J M, Czuczman M S, Reeder C B, Haioun C, Polikoff J, Tilly H, Zhang L, Prandi K, Li J, Witzig T E (2013). Long-term follow-up of lenalidomide in relapsed/refractory mantle cell lymphoma: subset analysis of the NHL-003 study.. *Annals of oncology : official journal of the European Society for Medical Oncology / ESMO*.

